# Memory Color Effect Induced by Familiarity of Brand Logos

**DOI:** 10.1371/journal.pone.0068474

**Published:** 2013-07-10

**Authors:** Atsushi Kimura, Yuji Wada, Tomohiro Masuda, Sho-ichi Goto, Daisuke Tsuzuki, Haruo Hibino, Dongsheng Cai, Ippeita Dan

**Affiliations:** 1 Department of Information Environment, Tokyo Denki University, Inzai, Chiba, Japan; 2 Sensory and Cognitive Food Science Laboratory, National Food Research Institute, Tsukuba, Ibaraki, Japan; 3 Faculty of Science and Engineering, Chuo University, Bunkyo-ku, Tokyo, Japan; 4 Graduate School of Engineering, Chiba University, Chiba-city, Chiba, Japan; 5 Graduate School of System and Information Engineering, University of Tsukuba, Tsukuba, Ibaraki, Japan; University of Missouri-Kansas City, United States of America

## Abstract

**Background:**

When people are asked to adjust the color of familiar objects such as fruits until they appear achromatic, the subjective gray points of the objects are shifted away from the physical gray points in a direction opposite to the memory color (memory color effect). It is still unclear whether the discrepancy between memorized and actual colors of objects is dependent on the familiarity of the objects. Here, we conducted two experiments in order to examine the relationship between the degree of a subject’s familiarity with objects and the degree of the memory color effect by using logographs of food and beverage companies.

**Methods and Findings:**

In Experiment 1, we measured the memory color effects of logos which varied in terms of their familiarity (high, middle, or low). Results demonstrate that the memory color effect occurs only in the high-familiarity condition, but not in the middle- and low-familiarity conditions. Furthermore, there is a positive correlation between the memory color effect and the actual number of domestic stores of the brand. In Experiment 2, we assessed the semantic association between logos and food/beverage names by using a semantic priming task to elucidate whether the memory color effect of logos relates to consumer brand cognition, and found that the semantic associations between logos and food/beverage names in the high-familiarity brands were stronger than those in the low-familiarity brands only when the logos were colored correctly, but not when they were appropriately or inappropriately colored, or achromatic.

**Conclusion:**

The current results provide behavioral evidence of the relationship between the familiarity of objects and the memory color effect and suggest that the memory color effect increases with the familiarity of objects, albeit not constantly.

## Introduction

Memory color refers to the colors that are recalled in association with familiar objects. Hering [1] was the first to claim that all objects known to us through past experience are seen through the spectacles of memory colors. Indeed, we associate canonical colors with familiar objects, such as yellow with bananas, red with tomatoes, green with grass, and blue with sky.

The remembered colors of familiar objects are often different from the original ones because memory color tends to enhance the chromatic features of objects. In many cases, an increase in saturation and luminance is observed [2], [3], [4], [5]. For instance, Bartleson [3] examined the memory colors of ten familiar objects such as green grass, red brick, and beach sand. In his study, participants chose a color patch which seemed to best represent the color of the objects from an array of 931 Munsell color samples. The mean memory colors of the ten objects were compared with average measured chromaticities of the corresponding natural objects. The results showed that each memory color was not of the same chromaticity as the actual color of the object and tended to shift toward the dominant chromatic attribute of the object (e.g., green grass was memorized as greener, red brick was memorized as redder). Furthermore, the memory colors had more saturation than the original ones in eight out of ten objects. Pérez-Carpinell et al. [5] also found that the colorimetric shifts in memory depend on the colorimetric purity of a given object. The purity of the remembered objects increased for objects with high purity values, but decreased or remained unchanged for objects with moderate or low purities.

Several studies have also reported that memory color modulates our color perception of familiar objects. For example, Hansen, et al. [6] demonstrated that the visual perception of familiar objects is strongly influenced by memory color. Their participants adjusted natural chromatic fruit and vegetable pictures until they appeared achromatic on an isoluminant color space. The subjective achromatic points of these pictures shifted away from the physical achromatic point in a direction opposite in the color space to the normal color of the objects. For example, the observers adjusted the color of a banana, whose canonical color was yellow, to a slightly bluish hue in order for it to appear gray. This phenomenon suggests that the observers perceived fruit and vegetable images as slightly colored in their canonical color even when the target object was achromatic. This bias in perception was compensated for by their setting the fruit and vegetable colors slightly opposite to their canonical colors. Hansen et al. named these shifts of subjective gray point *the memory color effect* and proposed that perception of an object’s color was not determined by incoming sensory data alone, but was modulated by the prior experience of having seen the natural color of the object.

Although the nature and functions of memory color have been widely studied by using well-known objects, little is known about the relationship between the degree of familiarity of objects and the chromaticity shift in memory (i.e., whether the memory color effect of a familiar object is stronger than that of a less familiar object). Traditionally, the target objects used for measuring memory color have been chosen only from well-known natural objects or substances, such as fruits, vegetables, grass, flowers, sky, and skin. While there are discrepancies between memory color and the actual color of objects with which people have several visual experiences, it is still unclear whether the discrepancy between memorized and actual colors of objects is dependent on the familiarity of the objects.

Here, we examined the relationship between the degree of a subject’s familiarity with objects and the degree of memory color effect by using brand logos with various familiarity levels (high, middle, low). In this study, logos of food and beverage companies which were present in the Japanese market were chosen as target stimuli for the following reasons: (1) Consumers’ familiarity with logos can be controlled by the size and region of the companies’ markets. (2) Logos usually consist of a small number of colors on homogeneous textures. Furthermore, photographs of natural objects include several colors other than the canonical color (e.g., the photograph of banana usually includes not only yellow coat but also green head and brown spots). These non-canonical colors of objects may also affect the appearance of the objects by color constancy. Thus, logos which consist of one chromatic color and achromatic colors on homogeneous textures may be more tightly controlled stimuli for measuring the memory color effect.

In Experiment 1, we measured the memory color effects of logos which varied in terms of their familiarity to participants by using the modified version of the method of adjustment developed by Hansen et al. [6]. In their study, participants conducted two tasks that were run in separate blocks. In one task, participants were asked to adjust each target object to look gray (called achromatic setting), and in the other task, they were asked to adjust each target object to look its typical color (called typical setting). In the present study, we used only the achromatic setting task because participants who had never been exposed to less familiar logos could not set their typical colors. We used the chromaticity of the original colors of the logo in place of the typical setting while following Hansen’s model (see Table 1). If a memory color is peculiar to familiar objects as suggested by previous studies, we would expect to observe a memory color effect only in logos in the high-familiarity condition but not in logos in the middle- or low-familiarity conditions. On the other hand, if the memory color is gradually developed as a function of the objects’ familiarity, the memory color effect should increase as the familiarity increases.

**Table 1:** Logos used in this study.

Familiarity condition	ID	Main product/serviceof the company	Dominant hue	*Y*	*x*	*y*	Number of domestic stores1)
	H1	Café	Bluish green	5.34	.224	.381	898
High	H2	Restaurant chain	Orange	19.50	.589	.369	1,154
(HF)	H3	Beverage	Red	7.65	.628	.337	–
	H4	Beverage	Blue	3.57	.145	.077	–
	M1	Beverage	Red	12.90	.626	.337	–
Middle	M2	Restaurant chain	Red	15.80	.629	.337	103
(MF)	M3	Packaged food	Red	15.80	.629	.338	–
	M4	Restaurant chain	Blue	1.29	.143	.076	51
	L1	Café	Brown	2.92	.563	.383	8
Low	L2	Restaurant chain	Purple	1.92	.575	.323	39
(LF)	L3	Supermarket	Red	15.70	.629	.337	–
	L4	Seafood industry	Light blue	11.70	.160	.142	–

1) The published number of domestic stores in Japan as of April 2011.

In addition, we elucidated whether the effect of the canonical color of logos relates to consumer brand cognition, such as semantic association between a brand and its food/beverage products, in Experiment 2. We assessed the semantic association between logo and food/beverage names using a semantic priming task [7], [8], [9]. This task is based on the spreading activation theories, which assume that presenting a related prime before the target lowers the activation threshold [7], [10]. In the semantic priming task, we primed the participants with the appropriately colored, inappropriately colored, or achromatic logo images used in Experiment 1. Immediately after priming, we presented them with food/beverage names as target stimuli and let them decide whether the name given was a food or beverage. We assumed the priming effect of an appropriately colored brand logo on the response time of the categorization as evidence of a strong connection between the color, logo and target food/beverage in semantic memory. By so doing, we aimed to estimate the semantic association between the color, logo and key product of the company.

## Materials and Methods

### Ethics Statement

The research followed the tenets of the Declaration of Helsinki. Written informed consent was obtained after a complete explanation of the study. The study was approved by the institutional ethics committee of the National Food Research Institute and the Tokyo Denki University.

### Experiment 1

#### Participants

Fourteen Japanese undergraduate and graduate students living in the Kanto region of Japan, 7 females and 7 males with an average age of 21.1 years (*SD* = 0.91) participated in this experiment. They were unaware of the experimental purpose, and were paid. Each participant had normal or corrected-to-normal visual acuity, and passed the Ishihara Color Test to screen out achromatopsy [11].

#### Stimuli

**Figure 1:**
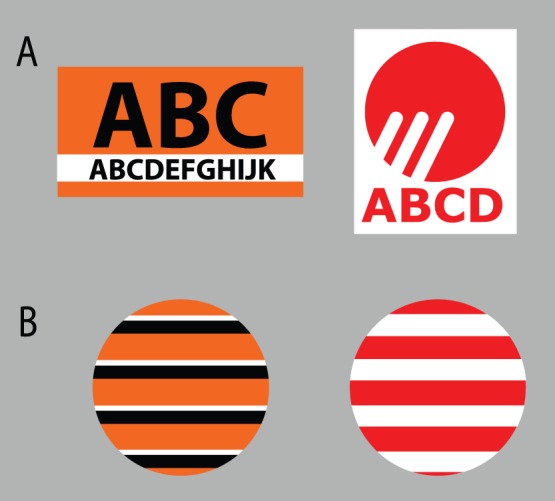
Schema designs for the logo stimuli (A) and the control stimuli (B).

We conducted a preliminary survey to select logos for high-familiarity (HF), middle-familiarity (MF), and low-familiarity (LF) conditions from a list of 24 possible logos. These logos were selected based on the following criteria: (1) The logos were from food or beverage companies which were present in the Japanese market; and (2) The colors of the logos consisted of one chromatic color and achromatic colors on homogeneous textures. Seventy-one students from universities in the Kanto region of Japan, 40 females and 31 males with an average age of 20.5 (*SD = *2.20), were asked to rate how often they saw each of 24 food or beverage company logos in their daily lives on a 5-point scale (1: “never” to 5: “always”). Based on the results of these familiarity ratings, the four most familiar logos (whose average scores of familiarity were closest to 5.0), the four most moderately familiar logos (closest to 3.0) and the four most unfamiliar logos (closest to 1.0) were selected as target stimuli for the HF, MF, and LF conditions, respectively (Table 1). Most companies with logos in the HF condition offer their products or services throughout the country. On the other hand, most companies with logos in the LF condition offer little their products or services only in the Kanto region. Each familiarity condition differed significantly on the scores of its familiarity rating, *F* (2, 140) = 586.46, (*p*<.01). Further *post-hoc* statistical analysis (Tukey’s HSD tests) indicated that the familiarity of logos in the HF condition (*M* = 4.92, *SD = *0.16) was higher than that of those in the MF (*M* = 2.96, *SD = *0.82) and in the LF (*M* = 1.81, *SD = *0.55) conditions (*p*<.01). Also, the familiarity of logos in the MF condition was higher than that of those in the LF condition (*p*<.01). The outer shapes of logos, such as square, rectangular, or circular, varied across target stimuli (Figure 1A). As control stimuli, we used horizontally striped disks whose color-to-area ratios were the same as those for each logo (Figure 1B). We used the DKL color space following the experiment in Hansen et al. [6]. The DKL color space is spanned by a luminance axis (L+M) and two chromatic axes (L – M and S – (L+M)) that define the isoluminant plane [12].

#### Apparatus

We used a personal computer (Dell Dimension 8300) with a 19-in. monitor (EIZO Flex Scan T766, special resolution of 800×600 pixels and refresh rate of 60 Hz) to present stimuli. Stimuli were presented on the monitor using Mathworks Matlab 2007b with a Cambridge Research VSG 2/5 graphics board. The size of the control stimuli and the average size of the target objects were 3.0 cm×3.0 cm (2.0 ×2.0 arc deg). The viewing distance between monitor and chinrest was 84.0 cm. The images were displayed on a mid-gray background, *x* = .259, *y* = .283 with a luminance of 29 cd/m^2^.

#### Procedure

The experiment was run in a dimly lit room to reduce visual distractions. Each participant was asked to binocularly observe the presented stimuli. In each trial, subjects were instructed to adjust the color of the target, which was presented in the chromatic color of the original logo, to look isoluminant gray. Participants adjusted the chromaticity in the isoluminant plane by pressing four keys that corresponded to the L – M and the S – (L+M) axes. Participants had no time limit for this task. When the participant was satisfied, their setting was recorded by the pressing of a key. After each trial, a 20-second inter-trial interval showing a blank screen was presented until the next trial. A total of 24 stimuli, which included 12 logos and 12 control stimuli, were presented randomly in a block. Each participant underwent 72 trials (24 stimuli×3 block repetitions). Participants conducted a short practice before the experiment. The results obtained in the practice session were excluded from further analysis. After completing the task of adjusting the color, participants were asked to complete a computer-based questionnaire about their subjective familiarity with each logo that had been presented. As the subjective familiarity rating, participants were asked to rate how often they saw each of the 12 logos in their daily lives on a 5-point scale (1: “never” to 5: “always”). The presentation order of logos was randomized across participants. When participants finished all experimental tasks, they were asked to complete a biographical questionnaire.

#### Data analysis

**Figure 2:**
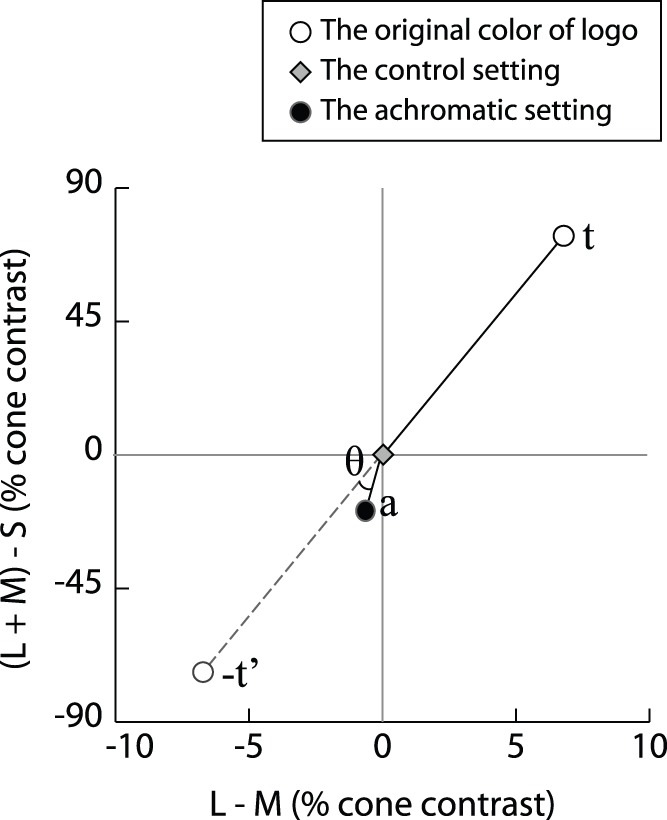
Schematic of analysis method for the memory color effect. This figure illustrates the quantification of the relationship between the magnitude and direction of the achromatic setting and the magnitude and direction of the original color. In this figure, a and t are the 2D vectors in the isoluminant plane for the achromatic setting and the original color of the logos. Both vectors were defined relative to each participant’s mean achromatic setting of the control stimuli.

Following Hansen et al. [6], we calculated the memory color index (*m*) scores (see formula below) as an index of the memory color effect for each logo. However, the coordinates of the typical settings in the equation used by Hansen et al. [6] were replaced by those of the original chromaticities of logos due to our experimental design. The relationship between the magnitude and direction of the achromatic settings to the magnitude and direction of the original colors were quantified as follows: The achromatic setting for each logo was projected on a line going through the original color and the achromatic setting for the control stimuli. The distance of this projected point from the control setting, divided by the distance of the original color from the control setting, was taken as an indicator of the strength of the memory color effect (Figure 2). Formally, the memory color index (*m*) was calculated using the following formula (1):
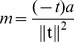
(1)where **a** and **t** are the 2D vectors in the isoluminant plane for the achromatic setting and the original color of the logos. Both vectors were defined relative to each participant’s mean achromatic setting of the control stimuli. If the achromatic settings aligned fully with the mirrored original color, the projection would correspond to the length of the achromatic vector. In this case, the *m* score would increase with the amplitude of the offset. On the other hand, if the achromatic setting were in a direction orthogonal to the original color, the projection would be near zero, independent of the amplitude of the achromatic setting, resulting in a low *m* score.

### Experiment 2

#### Participants

Seventeen Japanese undergraduate and graduate students living in the Kanto region of Japan, 6 females and 11 males with an average age of 21.7 years (*SD* = 1.27) participated in Experiment 2. Informed consent was obtained from each participant prior to the experiments.

#### Prime stimuli

**Figure 3:**
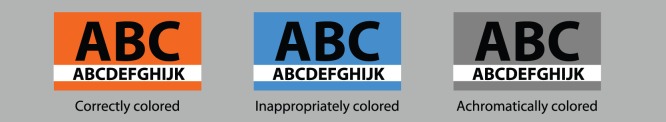
Illustrations for correctly, inappropriately colored and achromatic stimuli of the logo used in the semantic priming task.

We used the six kinds of logos used in Experiment 1 (H1, H2, H3, M3, L1 and L2; Table 1). The key products were coffee for the cafes (H1 and L1), and beef-rice-bowl for the restaurant chains (H2 and L2). We used H3 and M3 for dummy trials of the semantic priming task. The inappropriate (180° hue inversion) and achromatic pictures for each logo were created using the color adjustment function of Adobe Photoshop CS5 (Figure 3).

#### Target stimuli

Coffee and beef-rice-bowl (*gyu-don*) are the main food/beverage products of the brands which were included both in the HF and LF conditions. Thus, we aimed to compare the strength of semantic association between the logos and the names of the key food/beverages of HF brands with LF brands. Ten food-and-beverage names, including coffee and beef-rice-bowl, were used as target stimuli in the semantic priming task. The food/beverage names other than coffee and beef-rice-bowl were selected with a pilot study. In the pilot study, we had 43 participants (15 females; 28 males; average age = 20.1 years (*SD* = 1.46)) rate a list of 40 food and beverage names (20 each of food and beverages) for their liking and familiarity (5-point scale: 1 = *not at all* to 5 = *very*). The four food and four beverage names which had similar scores of liking and familiarity with those of beef-rice-bowl and coffee, respectively, were selected as the target stimuli. The selected food and beverage names did not differ in liking scores, *t* (42) = 1.43, *n.s.* (*M*_food_ = 4.2 (*SD* = 0.48); *M*_beverage_ = 4.1 (*SD* = 0.61)), or familiarity, *t* (42) = 1.42, *n.s.* (*M*_food_ = 4.0 (*SD* = 0.66); *M*_beverage_ = 4.2 (*SD* = 0.63)).

#### Procedure

**Figure 4:**
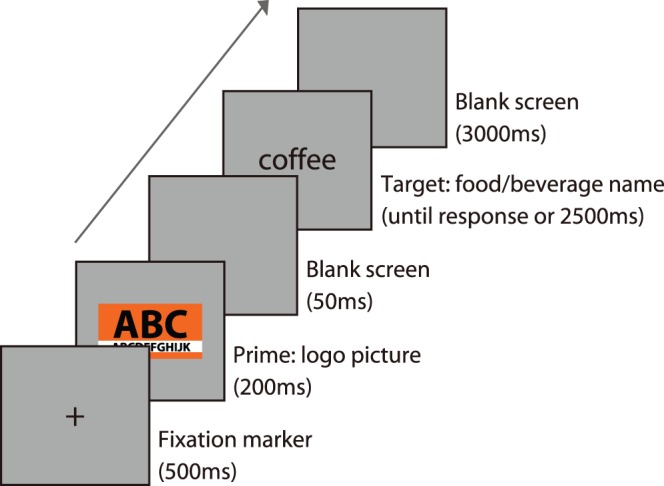
The time course of stimulus presentation in the semantic priming task.

Participants were recruited to take part in a word discrimination task experiment. The experiment was performed in small groups of up to five participants. Each participant sat in front of a 15-in. notebook computer monitor. The session consisted of four blocks, each of which consisted of 90 trials with an inter-trial time interval of 3000 ms, and an inter-block interval of approximately 2 min (for a total of 360 trials covering all possible parings of the 18 logo pictures×10 targets with 2 block repetitions). At the beginning of each trial, a fixation marker was presented for 500 ms. It was then replaced by the prime stimulus, which was presented for 200 ms. After a 50 ms inter-stimulus interval with a blank screen, the target stimulus was presented. Participants were requested to press the corresponding key (one for food and one for beverage) as quickly and accurately as possible upon judging whether the target name was a food or a beverage. The target stimulus remained on the monitor until a response was given or for 2500 ms when no response was given. After an inter-trial interval of 3000 ms, the next trial began (Figure 4). The order of stimulus presentation as well as which key was assigned to each (food or beverage) category was randomized across participants. The food/beverage choice and RT were recorded. Participants were informed that pairs of pictures and words would be sequentially presented at the center of the PC monitor, and were instructed to concentrate only on the second stimulus (food/beverage name). Prior to the session, the participants were given 12 practice trials to familiarize them with the experiment and the keys that they must press. After completing the semantic priming task, participants were asked to complete a questionnaire about their subjective familiarity with each logo that had been presented (only correctly colored ones). As the subjective familiarity rating, participants were asked to rate how often they saw each of the 6 logos in their daily lives on a 5-point scale (1: “never” to 5: “always”). When participants finished all experimental tasks, they were asked to complete a biographical questionnaire.

#### Data analysis

Each of the 17 participants performed a total of 360 trials. There were no non-response trials. Before analyzing the data, we discarded incorrect responses (e.g., a food name categorized as a beverage name), a total of 3.3% (202 trials) of all 6,120 trials. Of the remaining data, we only used the RT for the target name *coffee* for H1 and L1 logos, and the RT for the target name *beef-rice-bowl* for H2 and L2 logos in the following analyses. A separate 2 (logo familiarity, high vs. low)×3 (color of logo, correctly colored, inappropriately colored, or achromatic) repeated-measures ANOVA was performed on the RT for each of the two target names (coffee and beef-rice-bowl). Effect sizes (partial eta squared; *η*_p_^2^) were also calculated. When significant effects were detected, post hoc multiple comparisons of means were performed using Tukey’s honestly significant difference (HSD) test.

## Results

### Experiment 1

#### Validation of familiarity of logos

In order to examine the validity of the degree of familiarity of logos, a one-way repeated measures ANOVA with familiarity as a within-participant factor was performed on the subjective ratings of the familiarity of logos. Each familiarity condition differed significantly in familiarity scores, *F* (2, 26) = 227.16, (*p*<.01), *η*_p_^2^ = 0.95. Further *post-hoc* statistical analysis (Tukey’s HSD tests) indicated that the familiarity of logos in the HF condition (*M* = 4.9, *SD = *0.26) was higher than that of those in the MF (*M* = 2.7, *SD = *0.47) and in the LF (*M* = 1.7, *SD = *0.54) conditions (*p*<.01). Also, the familiarity of logos in the MF condition was higher than that of those in the LF condition (*p*<.01).

#### Effect of familiarity on the memory color effect (the *m* score)

**Figure 5:**
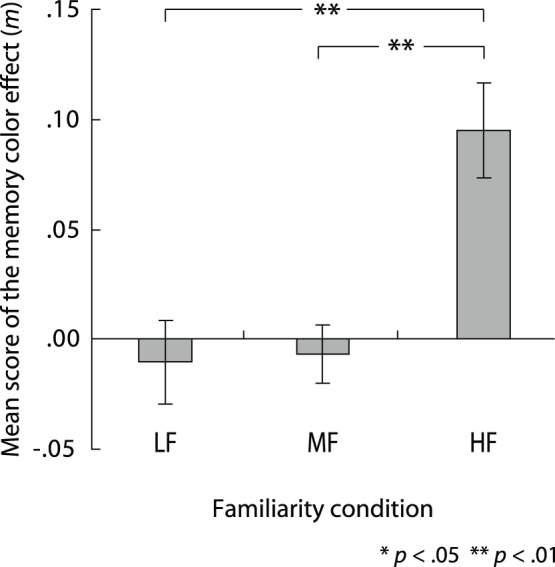
Effect of familiarity of logo on the memory color effect. Error bars indicate standard error (*N* = 14). Abbreviations are as follows: LF, low-familiarity condition; MF, middle-familiarity condition; and HF, high-familiarity condition.

The mean and standard errors for the *m* scores are presented in Figure 5. In order to examine whether the subjective gray points shifted away from the physical gray point in a direction opposite to the original color of the logos, two-tailed one-sample *t*-tests versus zero with Bonfferoni correction were performed on the *m* scores for each familiarity condition. The *t*-tests showed that under the HF condition the *m* score was significantly higher than zero (*t* (13) = 4.24, *(p*<.01)), while there was no significant difference from zero under the MF and the LF conditions. To determine whether familiarity affected the memory color effect, we conducted a one-way repeated measures ANOVA for the *m* score with the degree of familiarity as a within-participant factor. We found a significant main effect of the familiarity of a target (F (2, 26) = 8.74, *(p*<.01), *η*_p_^2^ = 0.40). Further *post-hoc* statistical analysis (Tukey’s HSD tests) among conditions indicated that the effect was higher under the HF condition than under the MF and the LF conditions (*p*<.01).

#### Effect of familiarity on the memory color effect in red logos

Supplementary analyses were conducted on the *m* scores of logos whose dominant colors were red in order to test whether the differences of chromaticity among logos used in this study were responsible for the *m* score. Logos with red targets were chosen for this analysis because they were included in each familiarity condition (H3 for the HF condition, M1–M3 for the MF condition and L3 for the LF condition; see Table 1). To examine whether the subjective gray points shifted away from the physical gray point in a direction opposite to the original color of the logos, two-tailed one-sample *t*-tests versus zero with Bonfferoni correction were performed on the *m* scores for H3, M1, M2 and M3 (averaged over the three), and L3, respectively. The *t*-tests showed that under the HF condition (H3; *M* = 0.14, *SD = *0.07) the *m* score was significantly higher than zero (*t* (13) = 7.76, *(p*<.01)), while there was no significant difference from zero under the MF (*M* = −0.003, *SD = *0.04) and the LF conditions (*M* = 0.09, *SD = *0.13).

#### Relationship between the memory color effect and the number of domestic stores of the brand

**Figure 6:**
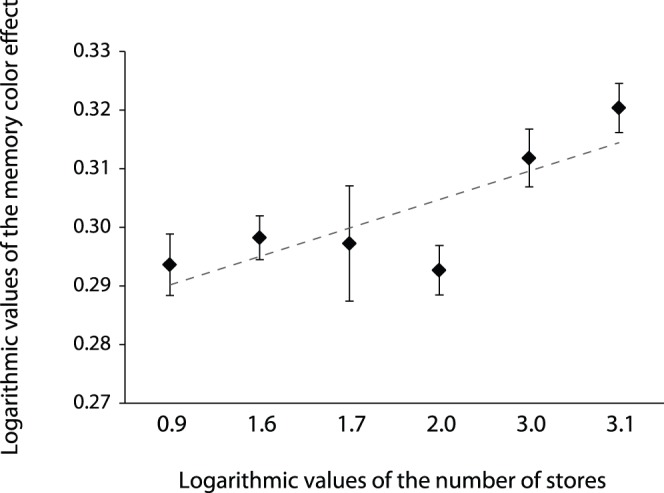
Correlation between the number of stores of the brand and the memory color effect. The logarithmic values were used both for the memory color effect and for the number of stores.

Further supplementary analyses were also conducted to examine whether the memory color effect related to the actual number of stores of the brand in Japan. Thus, we calculated the Pearson product-moment correlation between the amount of memory color effect and the number of stores in Japan for those companies that had actual stores (e.g., restaurant chain, cafe). In this analysis, the logarithmic values were used both for the memory color effect and for the number of stores. As a result, we found a significant positive and moderate correlation between the amount of memory color effect and the number of stores in Japan, *r* = .38, *p*<.05 (Figure 6).

### Experiment 2

**Figure 7:**
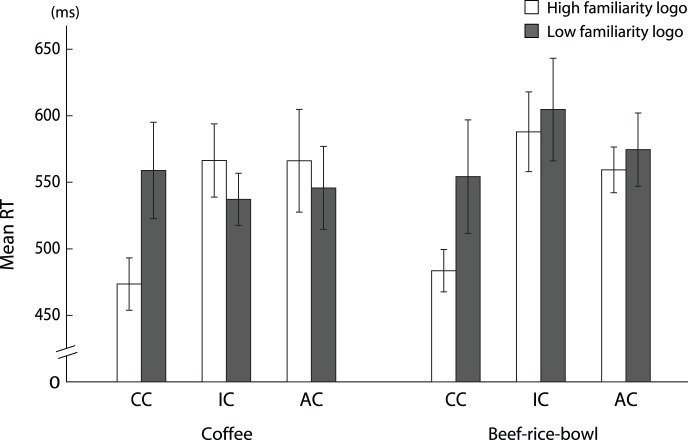
The mean RT (in ms) of the semantic priming task for coffee and beef-rice-bowl. Error bars indicate standard errors (*n* = 17). Abbreviations are as follows: CC, correctly colored image; IC, inappropriately colored image and AC, achromatic image.

The mean scores and standard errors for RT for coffee are presented in Figure 7. We found a significant interaction between logo familiarity and logo color, *F* (2, 32) = 4.88, *p*<.01, *η*_p_^2^ = .23. The tests of simple main effect showed that the RT for the correctly colored picture was shorter than those for the inappropriately colored and achromatic pictures of the HF logo (*p*s <. 05). On the other hand, we did not find any significant difference in RT among the three color conditions of the LF logo.

The mean scores and standard errors for RT for beef-rice-bowl are presented in Figure 7. A 2 (logo familiarity; high (H2) vs. low (L2))×3 (color of logo; correctly, inappropriately colored, or achromatic) ANOVA was performed on RT. We found a significant interaction between logo familiarity and logo color, *F* (2, 32) = 7.13, *p*<.01, *η*_p_^2^ = .36. The tests of simple main effect showed that the RT for the correctly colored picture was shorter than those for the inappropriately colored and achromatic pictures of the HF logo (*p*s <. 05). On the other hand, we did not find any significant difference in the RT among the three color conditions of the LF logo.

In order to examine the validity of the degree of familiarity of logos, we compared the subjective ratings of the familiarity of logos between HF (H1, H2) and LF (L1, L2) conditions by using a paired *t*-test. Results revealed a significant effect of logo familiarity, *t* (17) = 16.1, *p*<.01. The subjective familiarity of logos was higher in the HF condition (*M* = 5.0, *SD = *0.11) than in the LF condition (*M* = 1.8, *SD = *0.82).

## Discussion

The objective of the current study was to examine whether the participants’ familiarity with an object would affect the memory color effect. We measured the color appearance of logos of food and beverage companies with varying degrees of familiarity. The subjective gray points of the images shifted away from the physical gray point in a direction opposite to the memory color only in the highly familiar logos, but not in the moderately familiar and less familiar logos. The supplementary analysis using only logos with red targets confirmed this finding. Further analysis also demonstrated that there is a significant positive correlation between the memory color effect and the actual number of stores in Japan for the companies that have actual stores. These results indicate that the memory color effect differed depending on the familiarity of targets. The results of a manipulation check confirmed that the familiarity condition in our experimental design well reflected the subjective familiarity that participants held for the targets: Logos in the HF condition were reported as being more familiar to participants compared to logos in the MF and LF conditions. Logos in the MF condition were also reported as being more familiar to participants compared to logos in the LF conditions. Thus, our experimental results that the memory color effect emerges only with highly familiar logos were not explained by individual differences, but were due to the familiarity condition. These findings suggest that the degree of memory color effect increases with the familiarity of objects, albeit not constantly.

We found that observers consistently set the logo to a color off the gray point toward the opposite direction from the original color under the HF condition. Previous studies have noted that the memory color effect is observed in familiar objects. In most of these studies, natural objects and substances such as fruits, grass, trees, skin or sky were used as target objects. For instance, Hansen et al. [6] and Olkkonen et al. [13] found a significant memory color effect by using fruit and vegetable pictures as targets. The current results are consistent with previous findings which demonstrated that the memory color effect was evident in familiar objects, and further provide findings that this effect is evident not only in natural objects but also in artificial objects such as logos. As mentioned above, photographs of natural objects usually include not only the canonical color but also non-canonical colors of the objects (a yellow banana has a green head and brown spots, a red strawberry has a green hull and yellow seeds, and so on). Color constancy based on the whole relationship among the canonical and non-canonical colors may affect the color appearance of the object even if the canonical color of the object is shifted into achromaticity [14]. On the other hand, logos used in this study consisted of one chromatic color and achromatic colors on homogeneous textures, and therefore were resistant to the effects of color constancy on color appearance in the adjustment task. Thus, images of artificial objects such as logos may be more valid as targets than are photographs of natural objects as used in previous studies on memory color.

The memory color effect was observed in highly familiar objects, but not in moderately familiar objects. Several studies suggest that memory color is related to stabilization in or sharpening of long-term memory [3], [4], [5]. For instance, Newhall et al. [4] proposed that memory color was a selective result of the relative impressiveness of the various aspects of the original color in the object. More dominant, characteristic, and attractive aspects tend to be more impressive and more prone to survive in the memory. Furthermore, several studies on canonical color recognition have suggested that object-color association in the memory is not solely a function of learned association in visual experience, but is mediated by verbal codes [12], [15], [16]. For instance, Mitchell et al. [16] showed that children skilled at identifying the canonical colors of objects appear to use the verbal association between the object name and the color name to choose the correct visual choice. Gleason et al. [17] also demonstrated that the canonical color choice in 2- to 5-year-old children was partly predicted by children’s color-labeling skills. These findings imply that the memory shift of colors of familiar objects seems to be a result of elaboration of the typical color of the object by the repeated exposure of an object with attention to and verbalization of its (typical) color. Thus, the memory color may not emerge for objects which have been experienced only a few times. While it is still unclear how much exposure is necessary for objects to evoke their memory color, the memory color effect may not arise in moderately familiar objects but may be peculiar to highly familiar objects.

One may argue that there is a possibility that color-related biases were responsible for the degree of memory color effect, because the original colors of logos were not the same among familiarity conditions. The logos used in the present study were selected through a preliminary survey according to the following criteria: (1) The logo should be of a food or beverage company present in the Japanese market; and (2) The color of the logo should consist of one chromatic color and achromatic colors on homogeneous textures. As a consequence, the hue and purity of the colors in logos were different among familiarity conditions (see Table 1). Previous studies suggest that the effect of memory color differs according to the hue of the object. Pérez-Carpinell et al. [5] demonstrated that the shifts that were produced in the dominant wavelength with memory were observed in orange and red tomatoes, but not in green watermelons, yellow lemons, pink roses or purple eggplant, in the case of untrained observers with illuminant D65. Olkkonen et al. [13] also found that the strength of the memory color effect depended on the kind of fruit. The effect was largest for yellow fruits such as the lemon and the banana, and was weakest for the strawberry. These phenomena imply that there are hue-related biases in memory color, although which hue has a greater effect differs among studies. However, supplementary analysis demonstrated that the degree of memory color effect was dependent on the familiarity condition even if the logos of each condition had similar chromaticity. Thus, our results cannot be explained according to such hue-related biases in memory color. Rather, our results suggest that the memory color effect differed depending on the degree of familiarity of the logos. To elucidate the relationship between the familiarity and the memory color effect, further experiments with tightly controlled stimuli in chromaticity are required.

In addition, the present study employed a subjective rating task for measuring the familiarity of logos. While the results of the manipulation check and the correlation between the memory color effect and the actual number of stores confirmed the effectiveness of the familiarity condition, the actual frequency of contact with each logo was not measured in this study. Thus, we cannot be certain whether participants’ ratings of familiarity reflect their actual experience with logos. This prevented us from determining which factors in our experimental settings led to the current results. To make clear the degree of memory color effect as a function of an object’s familiarity, a further study, using targets which are controlled both in familiarity and in the frequency of contact with participants, is necessary. A promising strategy would be to use hypothetical new logos as targets, resulting in a controlled situation in which all brand- and logo-related inferences made by the participants would be based on exposure to marketing communication provided by mock advertisements. Such an experimental setting was used in a study evaluating new brand names [18], and may enable a controlled opportunity for participants to experience logos.

We also found that the degree of memory color effect relates not only with the subjective familiarity of logos but also with the actual number of stores. It is believed that if people have more opportunities to see stores and signs of a certain company, they may feel familiar with the logo of that company. These findings suggest that the memory color effect may reflect the degree of consumer brand cognition. To elucidate the relationship between the color of a logo and brand cognition, we measured the semantic association between a logo with appropriate coloring and the key food/beverage products of the corresponding brand using a semantic priming task in Experiment 2. For this task, it was hypothesized that the priming effect of an appropriately colored logo on the RT of the subsequent target judgments of the product was evidence of strong association between color, logo and target product in semantic memory. Results of Experiment 2 demonstrate an interactive effect of logo familiarity and logo color on a consumer’s semantic associations between logos and food/beverage names. The semantic associations between logos and food/beverage names in the high-familiarity brands were stronger than those in the low-familiarity brands only in the correctly colored condition, but not in the inappropriately colored and achromatic conditions (Figure 7). These results suggest that brand cognition, such as the association between logos and products, is determined not by the shape of the logo alone, but by the combination of color and shape at least in the brands which have logos that include one chromatic color with achromatic colors. These findings imply that the color of a logo strongly relates to consumer brand cognition, and therefore the memory color effect may be useful for assessing the advertising effects of the brand. It would be worthwhile to consider the possibility of industrial and marketing applications of visual characteristic such as the memory of color. Strictly speaking, the semantic association measured by this semantic priming task may reflect not only semantic association but also instrumental association. Recent theories of implicit social cognition discuss multiple processing of implicit cognition in relation to different implicit memory systems including semantic and instrumental memories [19]. Semantic association refers to links between concepts whereas instrumental association refers to repeated stimulus-action parings [19]. From this perspective, it is considered that the association learned through repeated pairings of logo and product name by advertisements and other media is semantic association. On the other hand, frequent users of the brand may also have an instrumental association learned by repeated pairings of logo and actual products of the brand. Thus, the semantic association between a brand and its products assessed in Experiment 2 might be a combination of semantic memory and instrumental memory. Further experiments tightly controlling the participants’ usage level of brands would allow elaboration of this discussion.

In conclusion, the current results provide the first behavioral evidence of the relationship between the familiarity of objects and the degree of memory color effect. Although there are some limitations, as mentioned above, these findings could serve as an important step towards understanding the nature of memory color. Further research is necessary to clarify how repeated exposure to an object leads to its memorization as a familiar object, and relates to the emergence of memory color.
